# Comparative evaluation of *BMI-1* proto-oncogene expression in normal tissue, adenoma and papillary carcinoma of human thyroid in pathology samples

**DOI:** 10.1186/s13104-021-05771-w

**Published:** 2021-09-22

**Authors:** Mohadeseh Hajian, Abolghasem Esmaeili, Ardeshir Talebi

**Affiliations:** 1 Department of Pathology, School of Medicine, Isfahan University of Medical Sciences, Isfahan, Iran; 2 Department of Cell and Molecular Biology, University of Isfahan, Isfahan, Iran

**Keywords:** *BMI-1* gene, Thyroid, Papillary carcinoma, Real-time PCR

## Abstract

**Objective:**

Papillary Thyroid carcinoma accounts for more than 60% of adult thyroid carcinomas. Finding a helpful marker is vital to determine the correct treatment approach. The present study was aimed to evaluate the expression of the B cell-specific Moloney murine leukemia virus integration site 1 (*BMI-1*) gene in papillary carcinoma, adenoma, and adjacent healthy thyroid tissues. Pathology blocks of thyroid tissues at the pathology department of patients who have undergone thyroid surgery between 2015 and 2019 were examined; papillary carcinoma, adenoma, and healthy tissues were selected and sectioned. Total RNA was extracted, and the relative expression level of the *BMI-1* gene was examined using the Real-Time qPCR method.

**Results:**

In the papillary and adenoma tissues, *BMI-1* was overexpressed (1.047-fold and 1.042-fold) in comparison to healthy tissues (p < 0.05 for both comparisons). However, no statistically significant differences were observed between adenoma and papillary carcinoma tissues regarding *BMI-1* gene expression. This study demonstrated a new biomarker for thyroid malignancies and found that the mRNA levels of the BMI-1 gene were higher in tumor tissues compared with healthy tissues. Further studies are needed to evaluate the *BMI1* gene expression in other thyroid cancers.

## Introduction

Although thyroid cancers represent only 1% of total diagnosed tumors annually, it is still the most common endocrine glands’ malignancy [1]. According to recent reports, thyroid cancers account for 2.3% of new cancer cases in Iran [2]. WHO studies have revealed that thyroid cancers are far more common among women (230,000 new cases annually) compared with men (70,000 new cases annually) [3]; moreover, thyroid cancers are the 7th commonly diagnosed malignancy in women and the 14th commonly diagnosed malignancy in men among Iranians [4, 5].

Thyroid cancers’ etiology has been investigated in numerous studies. Some contributing factors to thyroid cancer include exposure to ionizing radiation, goiter and benign nodules/adenomas, lifestyle (smoking and dietary habitats), and exposure to toxic chemicals [6, 7]. Thyroid carcinomas are classified into four main categories: Papillary, Follicular, Medullary, and Anaplastic carcinomas. The first two types of thyroid cancer account for more than 90% of the cases and have a higher treatment rate [8]. Although disease-related mortality of papillary thyroid carcinoma occurs mainly in patients in stage IV of papillary thyroid carcinoma, these patients represent a minority of patients [9].

Early diagnosis and treatment of thyroid tumors may prevent the involvement of the cervical lymph nodes [10, 11]. It has been reported that neck lymph nodes are involved in 46% of papillary thyroid carcinomas at initial diagnosis, though the proper treatment leads to long-time survival [12]. Even though advanced laboratory diagnosis of cancers has improved the technological ability for earlier diagnosis of silent thyroid tumors, the need for predictive markers is critical for selecting individuals with increased risk of thyroid carcinomas [13].

Recent studies have reported that B cell-specific Moloney murine leukemia virus integration site 1 (*BMI-1*), a transcription factor involved in regulating cell cycle and apoptosis, is upregulated in various cancers and related to poor prognosis [14, 15]. Interaction of *p16* (a tumor suppressor encoded by the *INK4a/Arf* locus) and *BMI-1* contribute stem cell-like features to cancerous cells and alter the biological behavior of tumors [16]. In addition, down-regulation of *BMI-1* in breast cancer cells inhibited cellular invasion and proliferation [17]. Several studies have suggested the overexpression of the *BMI-1* gene as a predictive marker of cancer initiation [18, 19]. Moreover, upregulation of *BMI-1* has been linked to tumor relapse, metastasis, and resistance to therapy in multiple human cancers [20].

Consequently, the present study is aimed to investigate the level of expression of the *BMI-1* gene in human adenoma, papillary thyroid carcinomas, and their healthy adjacent tissue samples. The present study is the first report of the expression of BMI-1 in thyroid cancerous and healthy adjacent tissues to the best of our knowledge.

## Main text

### Materials and methods

#### Tissue sample preparation and ethical statements

Paraffin blocks of thyroid specimens surgically excised from 21 patients from 2015 to 2019 and achieved at the pathology department of Seied-o-Alshohada Hospital were used in the present study. Written informed consents were obtained from all patients before surgical procedures. Three specimen blocks were prepared for each patient, including papillary carcinoma, adenoma, and adjacent healthy tissues; the specimens were recovered, and 10 μM sections were prepared after slide microscopy observations and confirmation of thyroid papillary carcinoma diagnosis.

#### Inclusion and exclusion criteria

Collections of the archived paraffin blocks of surgical resection specimens of the pathology department of Seied-o-Alshohada hospital from 2015 to 2019 were examined in this study. Twenty-one cases met the inclusion criteria of this study; patients who were not simultaneously diagnosed with PTC and thyroid adenoma were excluded from the investigation. The clinicopathological characteristics of the patients are summarized in Table 1.

**Table 1:** Clinicopathological characteristics of patients with papillary thyroid carcinoma whose tissue blocks were sectioned in the present study

Characteristics	No (%)
Age at diagnosis (mean ± SD)	
> 45	19 (90.5)
< 45	2 (9.5)
Sex	
Men	15 (71.4)
Female	6 (28.6)
Family history	
Yes	2 (90.5)
No	19 (90.5)
Tumor stage	
I	17 (81)
II	3 (14.3)
III	1 (4.8)
IV	0
Tumor size (mm)	
< 5	4 (19)
> 5	17 (81)
Lymph node metastasis	
Yes	1 (4.8)
No	20 (95.2)

#### RNA extraction and cDNA synthesis

RNA extraction from biopsy sections was performed using the RNeasy FFPE kit (Qiagen, Germany). The quality and quantity of extracted RNAs were verified using NanoDrop 2000, and cDNA synthesis was carried out using 1 μg of DNAase-treated total RNA samples by QuantiTect Reverse Transcription kit (Qiagen).

#### Real-time RT-PCR

Real-time q-PCR was conducted in triplicate, using QuantiFast SYBR Green PCR Kit (Qiagen) on StepOne Plus real-time qPCR System (Applied Biosystems, USA) following the recommended protocol of the manufacturer. The *GAPDH* (Glyceraldehyde-3-Phosphate Dehydrogenase) gene was selected as the reference gene, and the following primers were used to determine the expression of *BMI-1*: F: 5ʹ-ATACTTCTCTGTTGCTACG-3ʹ and R: 5ʹ-TGCCATCTGATTCTTACAA-3ʹ. Relative gene expression levels were evaluated by the comparative ΔΔCT method as described previously [21]. The GAPDH Forward primer, GAAGGTGAAGGTCGGAGTC, and GAPDH Reverse primer, GAAGATGGTGATGGGATTTC, were used based on Yazdani et al. studies [22].

#### Statistical analysis

Data analysis was done using Graphpad Prism V 8.1 for windows. One-way analysis of variance (ANOVA) was used to investigate the significance of the difference in *BMI-1* expression levels between thyroid papillary carcinoma, adenoma, and adjacent healthy tissues followed by post-hoc Dunnett’s test. Furthermore, a two-tailed paired t-test was used to evaluate the difference in *BMI-1* gene expression between adenoma and papillary carcinoma tissues. p values below 0.05 were considered significant.

### Results

#### RNA extraction and cDNA synthesis

In this retrospective cross-sectional study, we sought to determine the expression level of the *BMI-1* gene in adenoma, papillary carcinoma, and adjacent healthy tissues. Total RNA was extracted from sectioned blocks using the RNeasy FFPE Kit, and NanoDrop and agarose gel electrophoresis examined the quality and quantity of extracted RNAs.

#### Real-time qPCR and statistical analysis

**Fig. 1:**
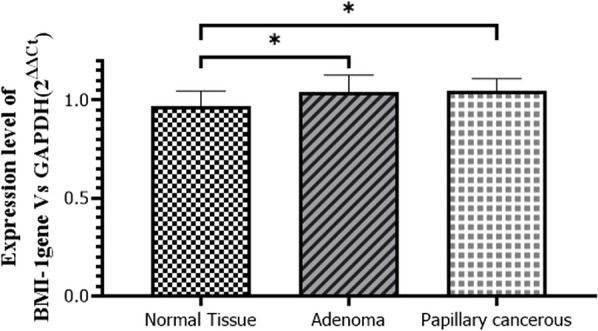
Comparison of expression of BMI-1 mRNA in papillary carcinoma, adenoma, and their adjacent healthy tissues. * p-values (0.0008) below 0.05 were considered significant

The expression level of the *BMI-1* gene in sectioned tissues was assessed using SYBR green real-time PCR assay. The results indicated that the *BMI-1* gene is slightly overexpressed in both types of tissues compared to their adjacent healthy tissues (Fig. 1).

Results of the Dunnett test revealed that the *BMI-1* gene was overexpressed in adenoma tissues by 1.042-fold (CI − 0.08533 to − 0.0003886, *p* = 0.04) and in papillary carcinoma tissues by 1.047-fold (CI − 0.09009 to − 0.005150, *p*: 0.02) compared with the adjacent healthy tissues.

Moreover, the results of the t-test indicated no significant difference between adenoma and papillary carcinoma in terms of the *BMI-1* gene expression level (*p* = 0.83).

### Discussion

Although conventional techniques for diagnosing thyroid carcinomas such as histological methods and FNA are considered gold standards, challenges in the differentiation of benign and malignant thyroid nodules remain unsolved [23–25]. Most thyroid carcinomas (differentiated papillary and follicular thyroid carcinomas) may have a promising prognosis and be treatable in case of timely diagnosis [26]. The early stratification of patients with poor prognoses would aid in selecting the most effective and appropriate therapeutic strategy [27, 28]. The lack of globally trusted biomarkers to determine aggressive types of thyroid cancer poses a significant challenge in managing and predicting patients with or at higher risk of thyroid cancer death [29].

In the present study, paraffin-embedded blocks of thyroid tissue specimens were evaluated from patients who underwent thyroid surgery from 2015 to 2019. Papillary carcinoma, adenoma, and a small part of adjacent healthy tissues were sectioned, and the expression level of *BMI-1* mRNA was measured in each tissue type. Comparison of *BMI-1* mRNA level in papillary carcinoma or adenoma tissues to adjacent healthy tissues revealed that *BMI-1* was slightly overexpressed in both tissues (*p* < 0.05 for both comparisons).

Several studies have documented that *BMI-1* overexpression is inversely correlated to the expression of tumor suppressor genes such as *PTEN* and *p16* [30]. Gisler et al. [31] have shown that upregulation of *BMI-1* significantly induces cancer cells proliferation. Furthermore, it has been shown that elevated BMI-1 mRNA level contributes to anti-cancer drugs resistance. Ojo et al. [32] reported that in breast cancer cell lines, *BMI-1* was upregulated, and knockdown of this gene sensitizes breast cancer cells to Tamoxifen, a widely used anti-cancer drug.

Some other studies reported the association between upregulation of *BMI-1* and the increment of differentiation but not proliferation. Dibenedetto et al. [33] reported that overexpression of *BMI-1* mRNA in myoblast cells correlates with elevation of mitochondrial activity and increases the energetic level of cells.

### Conclusion

In summary, the results of this study have shown that the *BMI-1* gene was upregulated in thyroid papillary carcinoma and adenoma tissues compared to adjacent healthy tissue. Although further studies are required to demonstrate the best thyroid cancer biomarker, these findings implicated that BMI-1 can be among candidates.

## Limitations

The main limitation of the study is the samples preparation. The blocks were found with adjacent normal tissues, and the patients’ consent to participate in the study.Also, BMI-1 proto-oncogene protein expression evaluation as gene transcripts are not provided to reflect the tissue protein expression due to the post-translational modifications and variable stability of the related mRNA.

## Data Availability

Please contact corresponding author (A.E.) for data requests.

## Abbreviations

*BMI-1*: B cell-specific Moloney murine leukemia virus integration site 1
FNA: Fine needle aspiration
*GAPDH*: Glyceraldehyde-3-phosphate dehydrogenase
PTC: Papillary thyroid carcinoma
*PTEN*: Phosphatase and tensin homolog
